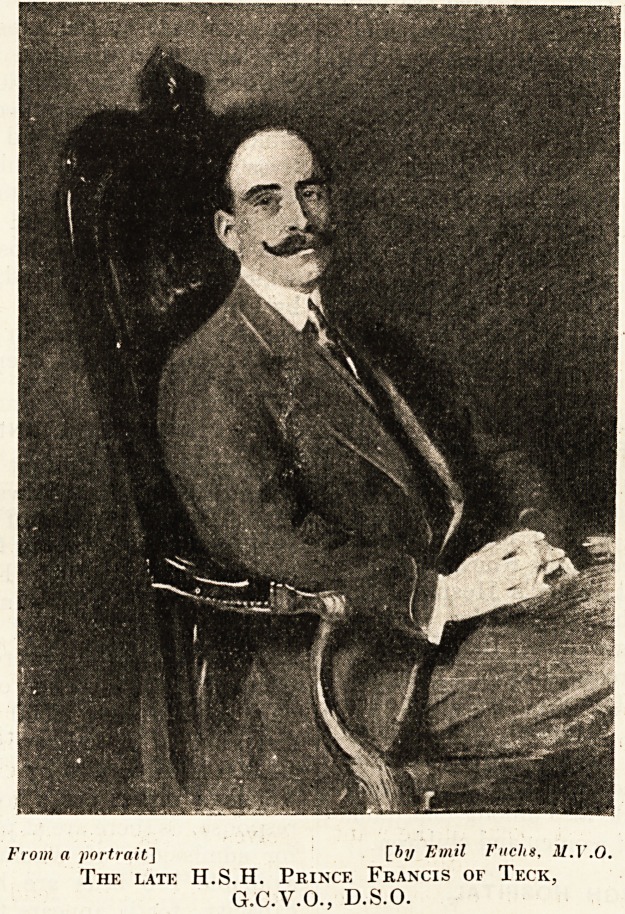# Hospital and Institutional News

**Published:** 1911-10-28

**Authors:** 


### HOSPITAL AND INSTITUTIONAL NEWS.

### EMULATE HIS EXAMPLE!

AMONG modern personalities few have had a greater charm or a better influence over others than the late Prince Francis of Teck. He has set an example to the younger generation of men which they might follow to their own uplifting and the advancement of England. Prince Francis's method was to think out and decide for himself whether a person or thing was good, and whether he cared to become more closely acquainted with it. If the decision was in the affirmative, he burnt his boats and devoted his whole energy to the cultivation of the friendship or the furtherance of the work. It was a great and fortunate day for Middlesex Hospital when Prince Francis of Teck decided to put himself at the head of its administration. Why do not many of the younger men up and down the country take up hospital work, follow the example of Prince Francis of Teck, and provide themselves with a life's motive, which will make them better and more useful men throughout the whole of their career? If any such need an object to work for, let them begin by sending their names to Prince Alexander of Teck, as voluntary workers in aid of the £200,000 Endowment Fund for Middlesex Hospital, which is to constitute the memorial to the late Prince Francis of Teck. Let the man and the object draw them.

### PRINCE FRANCIS OF TECK AND MIDDLESEX HOSPITAL.

No more touching or convincing enforcement of the advice just given could be forthcoming than the attendance and proceedings at a meeting, held on October 23, at the Middlesex Hospital of subscribers to a portrait of Prince Francis of Teck. We have not space to give the names of those present, but they represented great foreign nations, both Houses of the Legislature, the heads of the Church, of medicine, science, art, and, in short, of most that is best in the life of the nation. It is not the fashion to attend meetings of this kind nowadays, and the attendance referred to speaks more eloquently than language by pointing the moral of a young man's life well spent in the cause of those least able to help themselves. The Marquis of Ripon (unfortunately absent through illness) wrote that the subscribers to the portrait of Prince Francis were so numerous that £800 was received when only £300 was required. The balance of £500 was given as to one-half to the Middlesex Hospital and as to the other to the Hoxton Boys' Home. Lord Ripon, speaking with the knowledge of an intimate friendship, records of Prince Francis that he was as unstinting and generous in his friendships, as he was in all the good works which formed such a very important part of his life. Possessed of the most charming personality and essentially human, Prince Francis was the friend alike of the rich and the poor, the young and the old, and with his keen sympathy he shared equally in their sorrows and joys. Nothing lay nearer his heart than the Middlesex Hospital, for which with zealous energy and remarkable tact he raised £20,000. It was fitting that this portrait should be presented to and hung on the walls of the hospital he loved so dearly, for which he worked so untiringly, and where he was so universally beloved. Prince Alexander of Teck, who succeeded his brother as chairman, was evidently touched by the presence of so many friends who knew his brother's vivid personality, his charm of manner, and his unfailing strength of purpose. Prince Alexander well said the feelings of affection which have prompted the gift are in themselves a memorial to which the picture will be a tribute. He had the two-fold duty of handing over the portrait to the hospital as the representative of his family, and of accepting it gratefully on behalf of the Middlesex Hospital, of which he is the chairman. Through the courtesy of the artist, Mr. Emil Fuchs, M.V.O., we are enabled to publish a photograph of the portrait,

*From a portrait*] [*by Emil Fuchs, M.V.O.*

THE LATE H.S.H. PRINCE FRANCIS OF TECK, G.C.V.O., D.S.O.

which, as a picture and a likeness, is admirable. It indeed proves the ability and apprehension of the painter to a degree seldom achieved by artists in the present day.

### NEW WING AT THE CUMBERLAND INFIRMARY.

A new wing which has been added to the Cumberland Infirmary as a memorial to King Edward VII. will be opened on October 28 by Lady Lonsdale. The ceremony will be attended by the Mayor of Carlisle and the Corporation, and Sir Robert Allison will give an address. The completion of this greatly needed extension reflects great credit on the Building Committee, and is a worthy example of the modern demands of hospital construction.

### ALTERATIONS AT THE RADCLIFFE INFIRMARY.

Extensive alterations to this Infirmary are now being undertaken and a temporary out-patients' department has been erected in the rear of the main building for use while the rebuilding is in progress. The out-patients' department is being practically entirely reconstructed by Messrs. Brind, Gillingham and Co. Six rooms for the medical staff have been planned on the western side of the main building and new lavatories have been erected. A smart piece of work was the construction of the temporary building in three weeks—a very, creditable performance considering the circumstances and one reducing the dislocation of the work of this hospital to a minimum.

### LEGACIES TO HOSPITALS FOR WOMEN AND CHILDREN.

A large number of hospitals benefit under the will of Mrs. Charlotte Barnato, of Eastbourne. The London Hospital receives £1,000 for the female wards; £500 each is allotted to the women's wards at the Cancer Hospital, St. Mary's Hospital, the Samaritan Hospital, and the Hospital for Women, Soho Square; the children's wards at the Middlesex Hospital, the Paddington Green Children's Hospital, the Great Ormond Street Hospital, the Foundling Hospital, and the Jew's Hospital and Orphan Asylum also receive £500 each. Mrs. Barnato left the residue of her property, amounting to over £20,000, upon trust, to be divided among charitable institutions for the benefit of women and children.

### CROYDON BOROUGH HOSPITAL.

On October 23 the Mayor of Croydon formally opened the much-needed extension of the Borough Hospital at Waddon in the presence of many well-known local personages. The extension consists of two observation infectious pavilions, each containing twelve beds with plate-glass enclosures. Alterations have been made to the administrative block, and the nurses, who recently had to sleep in their wards, owing to lack of accommodation, are now each provided with a bed-room. The kitchen has been remodelled and a new laboratory added. We hope to illustrate shortly a plan of the pavilion design, and also some interesting details of the nurses' bed-room fittings and furniture.

### CEYLON SORE MOUTH.

Dr. Philip Bahr, of the London School of Tropical Medicine, has been selected to proceed to Ceylon for an investigation into sprue, or Ceylon sore mouth. He will probably leave England in the early part of the coming year. The expenses of the expedition will be defrayed by the Ceylon Government and the Planters' Association. Dr. Bahr has lately returned from a sojourn of eighteen months in Fiji, where he has been engaged on an inquiry into filariasis and dysentery.

### NEW WARDS AT THE ROYAL COUNTY HOSPITAL FOR CHILDREN, LIVERPOOL.

The new wards at this institution are now almost entirely completed, and were thrown open to public view on October 14. The additional accommodation makes provision for seventy children, though it appears that at the present time sufficient funds are lacking to maintain the full number of 150 children. On the occasion of this public view Sir William Bowring unveiled an inscription over a cot which has been endowed in perpetuity by his relatives to commemorate his golden wedding. The Chairman of the hospital, Colonel Montgomery, made an eloquent appeal for an increased subscription list to enable the hospital to wipe out the debt which, it was anticipated, would amount to £3,500 by the end of the year. At present eighty children are being maintained in this country hospital in large open-air wards, overlooking the estuary of the Dee.

### AN APPEAL AND A CHALLENGE.

In order that the first object of the Cardiff City Memorial to King Edward VII. may be accomplished before the end of the present year and that an impetus be given to find the additional income required for the King Edward VII.'s Hospital, Cardiff (the Cardiff Infirmary), Colonel Bruce Vaughan has just issued a further appeal in which is incorporated a generous challenge from "A Glamorganshire Owner of Property." This is an offer to contribute £500 provided that nine others are prepared to do likewise, or a sum equivalent to £4,500 is subscribed by Christmas. Colonel Bruce Vaughan makes out a good case for an immediate response, as there are at present 939 patients waiting for admission, of whom 300 are children. The available 196 beds are being used beyond their capacity, for it appears that room has been made for 203 patients, so it is exasperating to know that the new wing, capable of accommodating 1,400 patients a year, could be placed in commission within a few weeks if only the income for maintenance were forthcoming. It is hoped that Colonel Vaughan's appeal will meet with the success it deserves, and it is encouraging to remember that in 1906 when "A Glamorganshire Owner of Property" issued a similar challenge the response was immediate and more than adequate. £1,000 was then offered if a further £6,000 were subscribed by the end of the year, *and by the end of November* over £8,000 was contributed. May the present challenge prove equally provocative!

### FLORENCE NIGHTINGALE MEMORIAL.

The article we published last week has elicited both sympathy and public interest, and we have to thank the Press generally for the generous support they are giving to our effort to secure the completion of this Memorial Fund. We have received a number of letters with remittances from £100 downwards. From the highest to the humblest gratitude is being displayed, and one letter from Rotherhithe offering personal service reminds us pleasantly of the fact. It is further gratifying to be justified in our belief that the governors, committees, and medical staff of many institutions for the sick would be willing to help, and that quite a number of them have already intimated their wish to co-operate in this way. We propose to publish the first list of contributions next week, and may in response to inquiries say that the co-operation of the smaller institutions anxious to contribute collectively £5 each, and of individual workers who wish to secure 10s. or £1 each, will be welcomed and gratefully received. Offers of co-operation and contributions should be addressed to the Editor of The Hospital, 29 Southampton Street, Strand, London, W.C. We cannot all raise or give £25 each, but we can all do something, and the sooner we set about it, complete this Memorial to Florence Nightingale, and make it worthy of her memory, the better.

### MR. MOMBER'S BEQUESTS.

The bequests of the late Mr. Albert Momber, of Magdalene College, Cambridge, will ultimately benefit a large number of hospitals, for a sum amounting to nearly £24,000 will, subject to the life-interest of the testator's father, be divided into sixty-six parts, of which seven parts are to be handed to the Governors of University College, London, upon trust to apply the income in maintaining a scholarship or scholarships for science; six parts to University College Hospital; four parts each to the London Hospital and the Queen Square Hospital, W.C.; three parts to St. George's Hospital; ten parts equally between the Lister Institute, Chelsea, the Marine Biological Research Association, Plymouth, the Cancer Research Institution, and the Tuberculosis Research Institution; two parts each to the Royal Sea Bathing Infirmary, Margate, the Hospital for Consumptives, Ventnor, the Royal Asylum for the Blind, Chelsea, the Home for Crippled Children, Chelsea, the Frimley Sanatorium in connection with the Brompton Hospital; one-half of a part each to Dr. Barnardo's Homes, the Malvern Rural Hospital, and the Royal Free Hospital, Gray's Inn Road.

### COVENTRY AND WARWICKSHIRE HOSPITAL.

The extensions to this hospital which are to form the King Edward VII. Memorial for the City of Coventry are now well in hand, but the Organising Committee engaged in raising the fund of £25,000 reports that for the year ending September 30 the total amount obtained has only reached £8,545. As was pointed out by the Chairman of the Hospital Committee, Mr. E. M. Iliffe, this sum, although at first sight somewhat disappointing in view of the total aimed at, represents one of the largest amounts subscribed by any provincial town as a memorial to the late King. The simultaneous appeals of the county fund and of the hospital have no doubt had a good deal of influence on this Memorial Fund, and we feel sure that the hopeful view taken by Mr. E. M. Iliffe of the generosity of the citizens of Coventry will not be falsified. An interesting disclosure was made at the recent meeting of the Fund Committee in regard to the result of an experiment made with collecting boxes on the tramcars of the city. The amount collected in these boxes was only just over two pounds in about seven months—a sum that shows what widely different results may be obtained by similar methods in different localities, for we believe that in Bristol this mode of collection resulted in the contribution of a very large amount of money in a comparatively short time.

### THE PROBLEM OF INEBRIETY.

The programme arranged by the Society for the Study of Inebriety for the coming session is an interesting one, which provides for the discussion of many of the aspects of the important problems with which the Society concerns itself. The opening lecture, which will be the fourth Norman Kerr lecture, is to be delivered by Professor Woodhead in the theatre of the pathological department at the Downing Street Medical School at Cambridge. Dr. Woodhead has chosen as his subject the interesting theme of "The action of alcohol on body temperature and on the heart." To institutional workers the question of alcohol must prove interesting not only because of the reasons which make the average individual interested in it, but, moreover, because of the fact that some hospital authorities are of opinion that the pendulum, which during the last twenty-five years has swung so much away from alcohol as a necessity in institutions, is gradually swinging back. Temperance men make a great point of the reduction in the alcohol bills of the large hospitals, but it is still doubtful whether this excessive economy in alcoholic stimulants and increased expenditure on other forms of stimulants, notably the expensive animal extracts, has not been overdone. The lecture takes place on Tuesday, November 14, at five o'clock in the afternoon, and tickets of admission may be obtained on application to the Secretary of the Society at 139 Harley Street, W.

### MEDICAL INSPECTION IN L.C.C. SCHOOLS.

The report presented to the Education Committee of the London County Council on the result of the medical inspection of children in the L.C.C. schools during 1910 discloses many interesting and important findings. It appears that 172,619 children were examined by the 114 medical men then on the staff, and that of these 32.6 per cent. received advice cards; taking the figures of the previous year as well, the proportion of approximately one-third of those examined is again the average number of children requiring some form of medical advice or treatment. Defective eyesight or disease of the eye accounted for 18,923 cases, or about 11 per cent., while there were found defects of the throat or nose in 29,927 cases, or over 17 per cent., and deafness or ear disease occurred in 9,499 cases, or 5½ per cent. The physique of the children has been made the subject of considerable research, the results of which are presented in the form of charts. The various groups shown on the charts correspond very closely with the three classes distinguished by the British Association. The correspondence of the result with the predominant occupation of the parents in each area is, with a few exceptions, remarkably close. The lower averages are found in the riverside districts, North Kensington, and in a central area extending from the Strand district to Bow and Bromley. Summarised, the general appearance of the children examined showed the following percentages: Those in a healthy condition, 46.7; those described as fairly healthy in appearance, 41.8; and those classed as unhealthy or in bad condition, 11.5.

### SMALL BEGINNINGS.

From the village of Lodie, some eight miles from Cambridge, came a small band of enthusiastic collectors to hand over the substantial sum of £12 4s., representing the result of their annual house-to-house collection in the village in aid of the funds of Addenbrooke's Hospital. This collection is in excess of the amount which Messrs. D. Webb, W. T. Ayers, and George Ayers, the collectors, obtained last year. The £12 4s. was obtained from 202 contributors out of a population of 650. The collection was initiated some thirty years at least ago by a band of about four village enthusiasts in the then hamlet of Lodie, whose sympathy had been aroused by the fact that in spite of all the benefits the poor of this hamlet received very little support was given to the hospital in return. These four enthusiasts set to work in organising a house-to-house collection, and their first year's labours enabled them to send about £2 10s. as a contribution to the hospital. This collection, which had such a small beginning, has continued regularly all these years until now, and has increased year by year until it has now exceeded £12 as above mentioned. Of the original founders of this collection one only survives in the person of Mr. W. Webb, who is as enthusiastic as ever, in spite of the fact that he is well-nigh four score years. It is only one of many illustrations of what can be accomplished from small and modest beginnings.

### A MODERN SANATORIUM AT ROMSLEY.

The sanatorium of which the construction has just been commenced at Romsley, about ten miles from Birmingham, on a site 800 feet above the sea-level, is to be dedicated to the memory of Sir William Cook. The plans were prepared by Mr. F. W. Martin, and will provide for a building facing south, with three wings, the staff block being in the centre. The two wings are placed at an angle in order to catch the maximum amount of sunshine, and will accommodate fifty patients. On the ground floor a terrace will be built where the patients may sun themselves in lounge-chairs. Behind this will be a corridor into which they can retire should the sun be too hot. On the first floor will be a *Liegehalle* for the men, a big open room over the doctors' apartments in the centre of the building, where they may take the air on lounges. There will be a reading-room for the men, a recreation-room for the women, and a large dining-hall, which may, when occasion requires, be used as a recreation-room. The building will have a plain stuccoed front and tiled roof, and the floors are to be constructed of hennebique fire-resisting concrete. It may be recalled that the erection of the institution on the Cotswolds gave Birmingham the distinction of founding the first municipal sanatorium for consumptives, and since then the establishment of a similar institution at Yardley made provision for fifty beds, so that when the Romsley building is completed 140 beds will be available for the treatment of consumptives. Sir William Cook's fruitful work on the Health Committee for the Birmingham Hospital Saturday Fund was eloquently referred to by the Lord Mayor of Birmingham when he laid the foundation-stone last Saturday week.

### PRECAUTIONS AGAINST PLAGUE.

A report has been issued by the Local Government Board on suspected cases of human plague in East Suffolk, and on an epizootic of plague in rodents which has occurred in the same country. It comprises, in addition to a report on the whole subject by Dr. Bulstrode, observations by Drs. Martin and Rowland, and a report on the pathological and bacteriological examination of rodents by Drs. Petrie and MacAlister. In the autumn of 1910, as readers of The Hospital are aware, the Board was notified of four cases of "pneumonic plague" at Ireston; the bacteriological diagnosis of these cases was not subsequently fully confirmed by inoculation tests; but, having regard to subsequent events, it is a reasonable inference that they may have been plague. As the inquiry proceeded it became clear that rodents were involved, and a further investigation soon showed that the rats over a somewhat extensive area were infected with plague. It seems highly probable that the plague was introduced by means of infected rats imported with foreign grain coming from plague-infected countries. The possibilities of the spread of plague from rats to man are much smaller in this country than in India, and the human cases in Suffolk occurred under conditions of domestic uncleanliness. Dr. Arthur Newsholme, in an introduction to the report, points out that the chief danger of spread of plague among human beings is that infection may spread from person to person, or indirectly by means of the human fleas which infest houses under uncleanly conditions. Apart from improvement of domestic sanitation, the most important precaution is that all cases of obscure disease simulating influenza, or "blood poisoning," and all cases of pneumonia in a suspected district should be regarded as being possibly plague, that means should be taken for obtaining a bacteriological diagnosis, and that effectual isolation should be secured pending the completion of this test.

### DEATH OF DR. HILLIER.

At a time which is critical for the medical profession it is a matter of deep regret that the House of Commons should lose the services of one of its few medical members. Dr. Alfred Hillier, whose death occurred suddenly on Tuesday last, took an active part in the debates on the Insurance Bill, and was preparing in the session just commenced to renew his strenuous work in safeguarding the interests of the profession during the further progress of this Bill. Dr. Alfred Peter Hillier was first returned to Parliament as member for the Hitchin Division in January 1910, and successfully held the seat in the Unionist interest at the last election. He had a very varied career for a medical man, and when only sixteen he went out to South Africa, where he served in the Kaffir War of 1878-79. On his return he studied medicine at the University of Edinburgh, graduating M.B. in 1882 and M.D. in 1884. South Africa soon drew him again, and he took a resident surgical appointment at the Kimberley Hospital, to which institution he eventually became honorary visiting surgeon. Dr. Hillier was President of the South African Medical Congress in 1893, and was for over three years in partnership with Dr. Jameson in Kimberley. He was a member of the famous Reform Committee of Johannesburg, and, like all the other members, was a political prisoner in Pretoria in 1895-96. On his release he came to Europe, and settled in London. He was recognised as a leader in the fight against consumption, being nominated by King Edward (then Prince of Wales) to represent this country at the Berlin Congress for the Prevention of Tuberculosis in 1899. His book on "The Prevention of Tuberculosis" is a standard work, and its successor, revised by Professor Koch, is one of the best of the kind on the subject. On South African affairs he was also a great authority, and he wrote most of the articles on the subject in the "Encyclopædia Britannica."

### THE LATE COLONEL WARBURTON.

The funeral of the late Colonel W. P. Warburton, formerly Superintendent of the Royal Infirmary at Edinburgh, took place on Saturday last at Brookwood Cemetery. The Board of the institution over which he had presided for so many years was represented at the ceremony by his successor in office, Sir Joseph Fayrer, Bart., M.D., F.R.C.S. Ed., while several other institutions and bodies in which Colonel Warburton had been interested, or with which he had been associated during his lifetime, were represented. Colonel Warburton, who had reached the age of sixty-eight years, died at his home at Lowestoft last week, whither he had retired after relinquishing his post at Edinburgh. He was the son of the Honourable James Warburton, of Prince Edward Island, Canada, and received his preliminary education at the Charlottetown College. Later on he came to Edinburgh, where, after a distinguished course in the medical school, he graduated M.D., taking also his Fellowship of the Edinburgh Royal College of Surgeons. In 1866 he passed into the Indian Medical Service, and was civil surgeon in the Punjab, where he had an extensive practice, being appointed surgeon to the Rajah of Kapurthala. In 1894 he was appointed Principal Medical Officer and Sanitary Inspector-Commissioner in Assam, and soon afterwards Inspector-General of Civil Hospitals in the United Provinces. In these capacities he did much useful service, interesting himself especially in hospital administration and management, so that he might have been said to have been the first authority on the subject in India. When he retired from the service in 1899 he was appointed honorary surgeon to the Viceroy, and was gazetted a Commander of the Star of India. When he returned to England his services were at once requisitioned by the Edinburgh Royal Infirmary—his training-school. His work here, up to his retirement last year, has already been sketched in these pages, and all that need be said is that his management largely contributed to make the Infirmary what it is to-day, the premier hospital in Scotland. Tactful, and at the same time a strict disciplinarian, admirably informed on all points concerning his business, indefatigable in his efforts to devote himself to the institution with which he was associated, his term of office was a highly successful one, and when he retired it was felt that Edinburgh had lost a hospital administrator whose place it would not be easy to fill. His connection with the hospital, it is to be hoped, will be commemorated by some tangible monument in the near future, but his best monument remains in the splendid record of institutional service which he has left behind him.

### NATIONAL INSURANCE BILL.

The House of Commons reassembled on Tuesday for the autumn session, and on the motion of the Prime Minister it was resolved to take all the time of the House for Government business. On Wednesday the Prime Minister submitted a motion, allocating time (twenty days) for the further stages of the National Insurance Bill. It was thought until a short time ago that the Government did not intend to restrict the discussions, but in view of the fact that the Committee still has seventy clauses as well as the schedule to consider, and that the Budget and other important Bills have to be proceeded with, it would hardly be possible to pass the Insurance Bill before the end of the year if the debates were not kept well in hand. The first seventeen clauses were dealt with in Committee before the adjournment, and such further amendments as are desired in the clauses affecting hospitals and medical practitioners will be considered at the Report stage. A number of amendments have already been promised by the Chancellor of the Exchequer, but up to the present he has not given notice of any amendments directly affecting the position of hospitals. The Chancellor has promised, however, that if anything in the Bill has the effect of depriving any persons, who at present have the right to dispense medicines, of the right to dispense under the Bill (subject to three years' experience) the Bill will be amended so as to prevent their being deprived of that right. It is stated that Mr. Lloyd George has come to terms with the friendly societies and that the concessions promised to the societies do not cause any encroachment upon the position taken up by medical practitioners; if this be correct much of the opposition will be removed.

The clauses of the Bill which still have to be considered by the Committee, with the exception of those relating to the composition and duties of the various administrative bodies, are not of such importance to medical practitioners and hospital authorities as are some of the earlier clauses. Thus clauses 18 to 31 are mainly of interest to friendly societies; clauses 32 and 33 relate to deposit insurance and make provision for insured persons who, being employed contributors, have not joined an approved society within the prescribed time, or who, having been members of an approved society, have been expelled therefrom and have not joined another approved society; clauses 34 to 38 make provisions as to special classes of insured persons, including married women, aliens, sailors and soldiers and young persons under 16; clauses 39 and 40 relate to financial matters; clauses 41 and 42 make provisions for the appointment of commissioners and an advisory committee; clauses 43 to 45 relate to the composition and duties of the local Health Committees; clause 46 empowers approved societies and local Health Committees to demand inquiries into causes of excessive sickness, &c.; clause 47 deals with the erection of sanatoria; clauses 48 to 59 relate to supplementary questions; clauses 60 to 81 deal solely with unemployment insurance, and clauses 82 to 87 relate to matters of a general character. There are also nine schedules requiring careful consideration.

### STRATFORD-ON-AVON HOSPITAL EXTENSION.

The new wing at this hospital was opened last week by Lady Jane Carleton. The new block has been added at an outlay of £2,800, and the extension provides increased accommodation for nurses, a consulting-room for the medical staff, two emergency wards, and a new post-mortem room and mortuary. The alterations also include the entire rearrangement of the hot-water supply and heating apparatus, and the placing of a sterilising-room next to the operating theatre. There has been a great increase of work in all the departments of the hospital, and an appeal was made at the opening ceremony for another thousand pounds to enable the debt on the extensions to be cleared off.

### ST. AUSTELL COTTAGE HOSPITAL.

The building of a new cottage hospital for St. Austell and district was inaugurated on October 17, by the laying of several foundation-stones at Trewoon, in the presence of a very large gathering. For some years the need of such an institution has been felt in this populous district, where clay works abound, and it is largely due to the untiring efforts of the Chairman, Mr. H. S. Hancock, and the Secretary, Mr. H. Hodge, that the scheme has been successfully carried through. Stones were laid by Viscount Clifden, Viscountess Clifden, Sir Francis Layland-Barratt, and others. The cost of the operating theatre, estimated at £250, will be defrayed by Sir Francis Layland-Barratt, who has also given 100 guineas to the funds; Viscount Clifden has contributed 100 guineas and Mr. F. B. Fortescue £200 to the general fund. The building when equipped is estimated to cost £2,700, and the tender of Mr. Corkeek, of Redruth, for the construction of the hospital, amounting to £1,931, has been accepted. The sums already received or promised amount to £2,405, and there is a very good prospect of the hospital being able to commence work free from debt. The working men and the clay merchants of the district have taken an active and practical interest in the scheme, and the secretary expressed the belief that no difficulty would be found in raising £400 a year for the maintenance.

### THIS WEEK'S DRUG MARKET.

Several important changes in the value of drugs have taken place during the week and a number of articles are attracting attention. Menthol is very much dearer; in fact the price has nearly doubled during the last fortnight, the advance being due to scarcity. Japanese peppermint oil is also dearer, and oil of aniseed and oil of cassia are advancing in value. Another article which has greatly increased in price is sugar of milk, which, owing to scarcity, is something like double its normal value. Oil of eucalyptus is considerably dearer and in good demand at the present moment. Other articles which have advanced in price are cardamoms, ipecacuanha, oil of male fern and the better qualities of Jamaica sarsaparilla. The position of opium is practically unchanged, and there have been no further advances in makers' quotations for morphine and codeine. Cod-liver oil still tends slightly lower in price, notwithstanding that the chief consuming season is approaching. As regards menthol, buyers should act with caution; at the present price, which, as stated above, is nearly double the price quoted little more than a fortnight ago, it is hardly advisable to buy more than is necessary for immediate requirements; the value may increase, but, on the other hand, menthol is such a deceptive market that there may be a substantial pull in price before long. As to sugar of milk, it is difficult to foresee what may happen, but it is not thought probable that any material reduction in value will take place immediately; the present, however, is not the time to buy in large quantities. Santonin has again been advanced in price, quotations now being abnormally high.

### A CORRECTION.

In last week's issue, by an oversight, Mr. Huyssen's medical education was ascribed to Guy's Hospital instead of to the London. As then announced, Mr. Huyssen has recently taken up the duties of resident medical officer at the Samaritan Free Hospital.

## Figures and Tables

**Figure f1:**